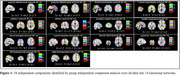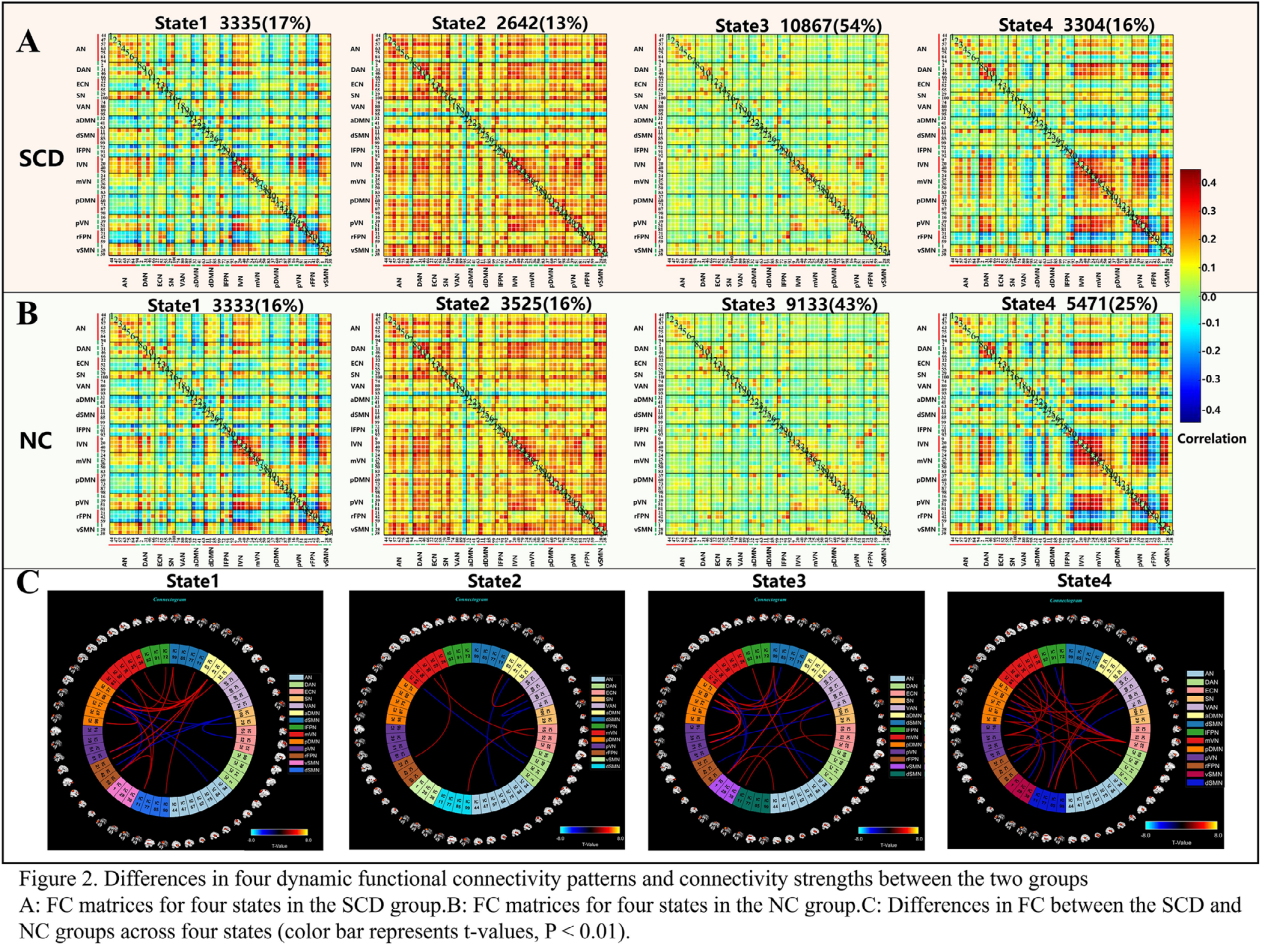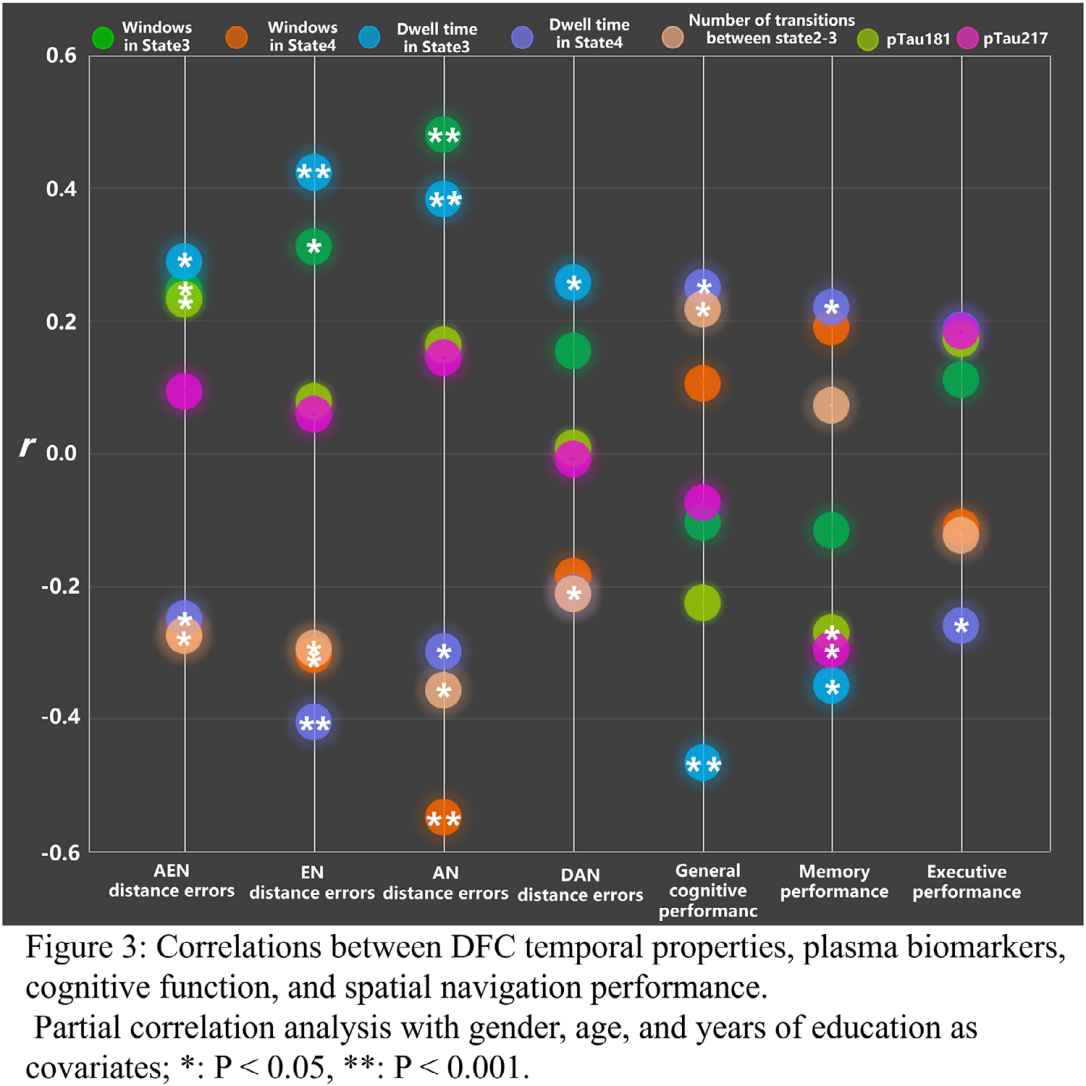# Dynamic Functional Connectivity Changes in Subjective Cognitive Decline and Their Association with Spatial Navigation: A 5.0Tesla Ultra‐High Spatiotemporal Resolution fMRI Study

**DOI:** 10.1002/alz70856_098138

**Published:** 2025-12-24

**Authors:** Futao Chen, Bing Zhang, Qian Chen, Xiang Fan

**Affiliations:** ^1^ Department of Radiology, Nanjing Drum Tower Hospital, Affiliated Hospital of Medical School, Nanjing University, Nanjing, Jiangsu, China; ^2^ Nanjing Drum Tower Hospital, Affiliated Hospital of Medical School, Nanjing University, Nanjing, Jiangsu, China; ^3^ Peking University Shenzhen Hospital, Shenzhen, Guangdong, China

## Abstract

**Background:**

Subjective cognitive decline (SCD) is considered the optimal window for early diagnosis and intervention in Alzheimer's disease (AD). SCD show changes in dynamic functional connectivity (DFC). Spatial navigation dysfunction is a sensitive biomarker for identifying SCD. However, the relationship between spatial navigation and DFC changes in the SCD is unclear. Ultra‐high field magnetic resonance imaging (HUF‐MRI) provides ultra‐high spatiotemporal resolution fMRI, making it possible to explore subtle functional changes in the brain in SCD. This study aimed to investigate the characteristics of DFC changes in SCD and their relationship with spatial navigation function and cognitive performance using 5.0T ultra‐high spatiotemporal resolution fMRI.

**Method:**

A total of 49 normal control (NC) participants and 46 individuals with SCD were recruited for comprehensive neuropsychological assessments, spatial navigation behavioral tests, plasma biomarker analysis, and ultra‐high spatiotemporal resolution fMRI scanning. Based on the sliding window method and k‐means clustering, distinct DFC states were identified. The number of windows, mean dwell time, and the number of transitions between each pair of DFC states were calculated. Finally, partial correlation analysis was used to assess the relationship between the temporal properties of DFC and spatial navigation function and cognitive performance.

**Result:**

Four distinct connectivity states were identified. Compared to NC, SCD exhibited more windows and longer mean dwell times in state 3, characterized by low connectivity both within and between networks. In contrast, in state 3, the SCD group showed fewer windows and shorter mean dwell times, dominated by high connectivity within the visual network and between the sensorimotor network, visual network, and attention network. Additionally, the number of transitions between state 2‐3 was significantly reduced in the SCD group. Correlation analysis indicated significant associations between DFC temporal properties with spatial navigation function and cognitive performance.

**Conclusion:**

The findings of this study suggest that ultra‐high spatiotemporal resolution fMRI using 5.0T MRI is an effective tool for investigating DFC changes in SCD. Alterations in DFC temporal properties in the SCD population may underlie early impairments in spatial navigation functions and can serve as sensitive neuroimaging biomarkers for the preclinical detection of AD.